# Dihydroartemisinin Modulates Apoptosis and Autophagy in Multiple Myeloma through the P38/MAPK and Wnt/*β*-Catenin Signaling Pathways

**DOI:** 10.1155/2020/6096391

**Published:** 2020-08-15

**Authors:** Xiuhua Wu, Yang Liu, Enfan Zhang, Jing Chen, Xi Huang, Haimeng Yan, Wen Cao, Jianwei Qu, Huiyao Gu, Ruyi Xu, Jingsong He, Zhen Cai

**Affiliations:** ^1^Department of Pharmacy, The First Affiliated Hospital, Zhejiang University School of Medicine, Hangzhou, Zhejiang, China; ^2^Bone Marrow Transplantation Center, Department of Hematology, The First Affiliated Hospital, Zhejiang University School of Medicine, Hangzhou, Zhejiang, China

## Abstract

Dihydroartemisinin (DHA), an active metabolite and derivative of artemisinin, is the most effective antimalarial drug and has strong antitumor activity in various tumor types. It has recently been reported that DHA can induce autophagy and has significant effects on multiple myeloma (MM), but the mechanisms and the relationship between the autophagy and apoptosis induced by DHA remain to be elucidated. Herein, we demonstrated that DHA significantly induces cell death in a dose- and time-dependent manner via the extrinsic and intrinsic apoptosis pathways. Moreover, DHA-induced autophagy, which plays a prodeath role in MM, can regulate canonical apoptosis and vice versa. Furthermore, the P38/MAPK signaling pathway is responsible for decreased autophagy and increased apoptosis. DHA induces autophagy and apoptosis also through the inhibition of the Wnt/*β*-catenin signaling pathway. In addition, DHA shows a strong effect in a xenograft mouse model. Collectively, these findings reveal that DHA, as an artemisinin-based drug, could be an effective and safe therapeutic agent for MM.

## 1. Introduction

Multiple myeloma (MM) is an incurable malignancy disease characterized by clonal plasma cell accumulation in the bone marrow accompanied by the secretion of monoclonal immunoglobulins in the serum and/or urine [1, 2]. In recent years, despite new treatment options, such as proteasome inhibitors, immunomodulatory drugs and monoclonal antibodies for myeloma patients, which have helped to enhance the median overall survival (OS), chemoresistance and recurrence remain major problems in the clinical management of MM [3]. Therefore, the development of new pharmacologically effective agents from natural compounds to induce MM cell death with minimal side effects would provide significant clinical benefits.

Dihydroartemisinin (DHA), a derivative and active metabolite of artemisinin isolated from the traditional Chinese herb *Artemisia annua*, has been widely used as a first-line antimalarial therapy [4, 5]. Emerging studies have shown that DHA also has strong antitumor activity in various tumor types by inhibiting cell proliferation, arresting the cell cycle, promoting apoptosis, and inhibiting angiogenesis and metastasis [6–9]. Furthermore, previous studies have reported that DHA induces autophagy and inhibits MM cell proliferation [10, 11]. However, the mechanisms underlying the effects of DHA in MM remain to be further investigated.

Macroautophagy (hereafter referred to as autophagy) is an evolutionarily conserved membrane-trafficking process. Intact organelles and portions of the cytosol are sequestered within double- or multimembrane vacuoles and finally delivered to lysosomes for degradation [12]. Autophagy is a double-edged sword in tumorigenesis. In some cellular settings, it can serve as a cell survival mechanism, and in others, it can lead to cell death, either in collaboration with apoptosis or as a back-up mechanism [13]. Although previous studies have shown that DHA induces autophagy in MM cells, whether autophagy actively induces cell death or is a cell response mechanism and the mechanisms underlying the apoptosis and autophagy induced by DHA are still unclear.

## 2. Materials and Methods

### 2.1. Cells

The human MM cell lines CAG, JJN3, and RPMI-8226 were a kind gift from Dr. Qing Yi (Center of Hematologic Malignancy Research Institute, Houston Methodist, Houston, TX). Primary CD138(+) cells were sorted using CD138 microbeads (Miltenyi Biotech, CA, USA). All cells were cultured in RPMI-1640 medium containing 10% fetal bovine serum and 1% L-glutamine at 37°C in a 5% CO_2_ atmosphere.

### 2.2. Cell Proliferation Assay

Cell counting kit-8 (CCK-8) assays were used to assess MM cell proliferation. MM cells (2 × 10^4^ cells/well) were seeded in 96-well plates and treated with the indicated concentrations of drugs at 37°C in a 5% CO_2_ atmosphere. After being incubated for the indicated lengths of time, the cells were treated with 10 *μ*l of CCK-8 solution for another 4 h, and then, the absorbance was measured at 450 nm using a microplate reader (Bio-Rad, Model 680).

### 2.3. Reagents and Antibodies

Hydroxychloroquine sulfate (HCQ), Z-VAD-FMK, and SB203580 were obtained from Selleck Chemicals LLC (Houston, TX). LiCl was purchased from Sigma-Aldrich, Billerica (MA, USA). Primary antibodies against caspase-3, caspase-8, poly(ADP-ribose) polymerase (PARP), P38, p-P38, *β*-catenin, *β*-actin, bax, and c-Myc were purchased from Cell Signaling Technology (MA, USA). Primary antibodies against CyclinD-1 and GAPDH were purchased from Abcam (Cambridge, UK). An anti-LC3B antibody was purchased from Sigma-Aldrich (Billerica, MA, USA). Caspase-9, Bcl-xl, and bad were purchased from Proteintech (Hubei, China).

### 2.4. Flow Cytometry

The cells were stained with Annexin V FITC and PI (Dojindo, Kumamoto, Japan) according to the manufacturer's instructions for analysis of apoptosis. Then, the treated cells were detected using a flow cytometer (BD Biosciences, San Diego, CA, USA) and analyzed using FlowJo X 10.0.7.

### 2.5. Immunohistochemistry

Tumor tissue samples from tumor-bearing NOD-SCID mice were fixed in 4% paraformaldehyde and embedded in paraffin before sectioning and were then used for immunohistochemical staining to analyze cleaved caspase-3.

### 2.6. Western Blot Analysis

Cells were collected and washed at least twice with PBS and lysed with RIPA lysis buffer containing protease and phosphatase inhibitors (Thermo Fisher Scientific). The supernatants were collected for western blot. Equal amounts of protein were separated by SDS-polyacrylamide gel electrophoresis (PAGE) and transferred to polyvinylidene difluoride membranes (Merck Millipore, Darmstadt, Germany). After being blocked with 5% nonfat milk or 5% bovine serum albumin for 2 h, the membranes were incubated with specific primary antibodies overnight at 4°C. The next day, the membranes were washed three times with Tris-buffered saline containing Tween 20 (TBST) and incubated with horseradish peroxidase- (HRP-) conjugated anti-rabbit or anti-mouse antibodies at room temperature for 1 h. The membranes were then washed three times with TBST, and images were collected using a ChemiDoc^TM^ MP Imaging System (Bio-Rad) and an Enhanced Chemiluminescence Detection kit (Biological Industries, Israel, Beit Haemek Ltd., Kibbutz Beit Haemek, Israel).

### 2.7. In Vivo Xenograft Studies

Approximately four-week-old male non-obese diabetic severe combined immunodeficiency (NOD-SCID) mice were obtained from Vital River Laboratory Animal Technology Co., Ltd. (Beijing, China) and then housed in the animal facility of Zhejiang University School of Medicine. CAG (1 × 10 [7]) cells were injected subcutaneously into the right flanks of the mice. After 11 days, when the established tumors reached approximately 100–130 mm^3^, the mice were randomly divided into two groups and administered intraperitoneal injections of DHA (10 mg/kg, every 3 days) or vehicle as a control for 10 days. The tumor size was monitored every 3 days with calipers, and the tumor volume was evaluated as 4*π*/3 × (*a*/2) [2] × *b*/2, where *a* is the tumor width and *b* is the tumor length. When the tumor volumes reached approximately 3,000 mm^3^, the mice were sacrificed. All experiments followed the procedures and protocols of the Animal Ethics Committee of the First Affiliated Hospital, Zhejiang University School of Medicine.

### 2.8. Statistical Analysis

All data are expressed as the mean ± standard deviation (SD) of at least three independent assays. GraphPad Prism 7.0 software (GraphPad Software, CA, USA) was used for all statistical analyses. Two-tailed Student's *t*-tests were used to determine the significance of differences between the experimental groups. *P* values less than 0.05 were considered significant.

## 3. Results

### 3.1. DHA Has Antimyeloma Effects

**Figure 1:**
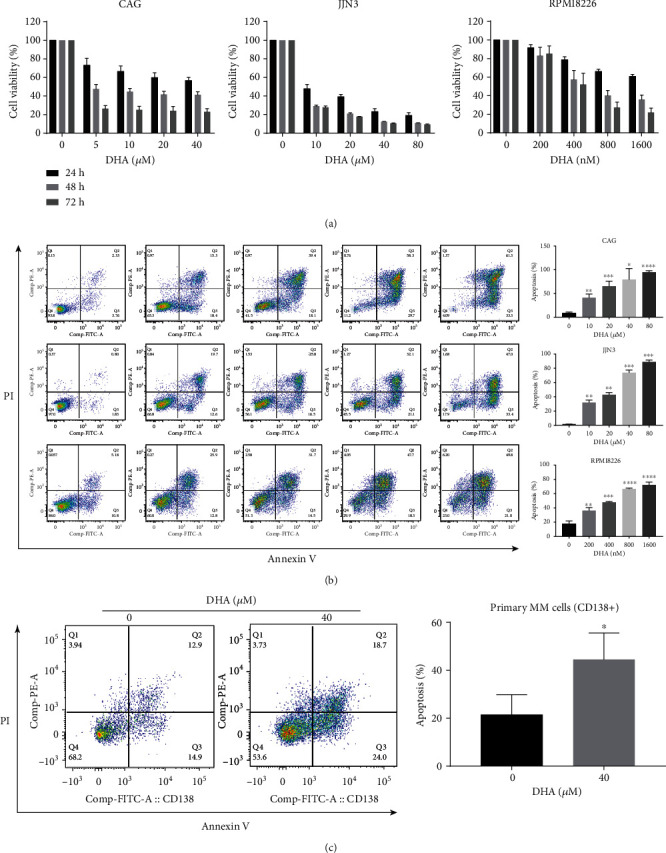
DHA has antimyeloma effects. (a) CCK-8 to test the inhibitory effect of DHA on MM cell proliferation. HMCLs were treated with different concentrations of DHA for 24, 48, and 72 h. (b) HMCLs were incubated with DHA or vehicle for 24 h and analyzed by flow cytometry. A representative flow cytometry analysis showing the apoptosis of HMCLs induced by DHA. The results summarizing at least three independent experiments are shown. Values are presented as the mean ± SD. (c) DHA treatment for 24 h significantly induces primary CD138+ plasma cell apoptosis (*n* = 3). Data are presented as the mean ± SD of at least three independent experiments. ^∗^*P* < .05, ^∗∗^*P* < .01, ^∗∗∗^*P* < .001, ^∗∗∗∗^*P* < .0001.

To investigate the potential inhibition of cell growth of DHA on MM, the human myeloma cell lines (HMCLs) CAG, JJN3, and RPMI-8226 were treated with increasing concentrations of DHA for 24 h, 48 h, and 72 h, respectively, and cell viability was evaluated by a Cell Counting Kit-8 (CCK-8) assay (Figure 1(a)). DHA treatment significantly decreased the myeloma cell viability in a dose- and time-dependent manner. We next examined the effect of DHA on apoptosis induction by Annexin V/PI staining-based flow cytometry at 24 h. Treatment of the HMCLs with DHA markedly increased apoptosis (Figure 1(b)). To further confirm these findings in primary MM samples, we measured apoptosis in CD138+ plasma cells and treated them with DHA or vehicle control for 24 h. Similar to our findings in HMCLs, DHA induced the apoptosis of CD138+ plasma cells from MM patients (Figure 1(c)). Taken together, these findings indicate that DHA reduces MM cell viability and promotes apoptosis.

### 3.2. DHA Induces Apoptosis through the Extrinsic and Intrinsic Pathways in MM Cells

**Figure 2:**
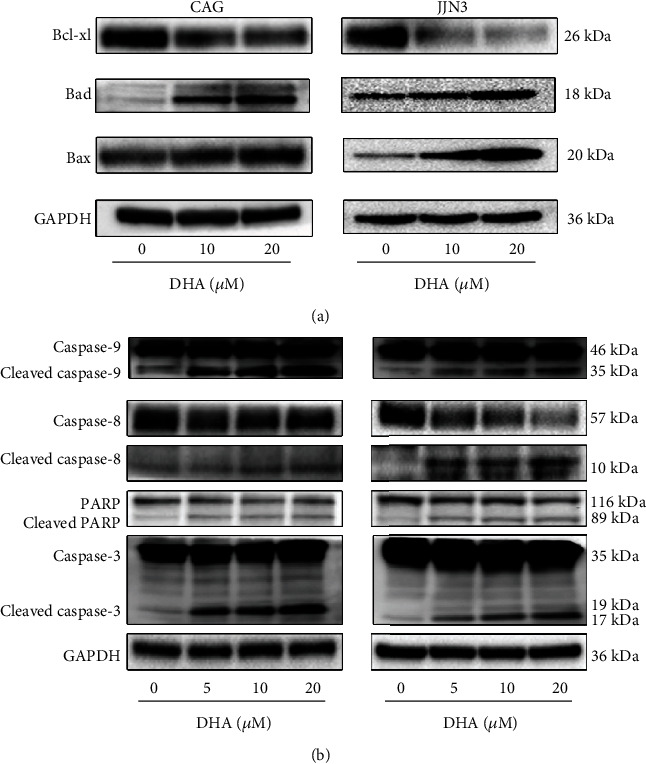
DHA induces apoptosis through the extrinsic and intrinsic pathways in MM cells. (a) HMCLs were exposed to various concentrations of DHA for 24 h. The expression of the following apoptosis-related proteins was determined by western blot analysis: Bcl-xL, Bad, and Bax. (b) HMCLs were exposed to various concentrations of DHA for 24 h. The expression of the following apoptosis-related proteins was determined by western blot analysis: caspase-9, caspase-8, PARP, and caspase-3. Data are presented as the mean ± SD of at least three independent experiments.

To explore the molecular mechanism by which DHA induces apoptosis in MM cells, we performed western blot to assess the expression of apoptosis-related proteins. As expected, the expression levels of Bad and Bax were dose-dependently increased in the DHA-treated MM cells at 24 h, and the expression of Bcl-xl was downregulated in the DHA-treated MM cells (Figure 2(a)). Moreover, we found that there were dose-dependent increases in the expression levels of cleaved caspase-9, cleaved caspase-8, cleaved caspase-3, and cleaved PARP in the cells treated with DHA at 24 h (Figure 2(b)). These results indicate that both the extrinsic pathway and the intrinsic pathway are involved in the antitumor activity of DHA.

### 3.3. The Connection between DHA-Induced Autophagy and Apoptosis

**Figure 3:**
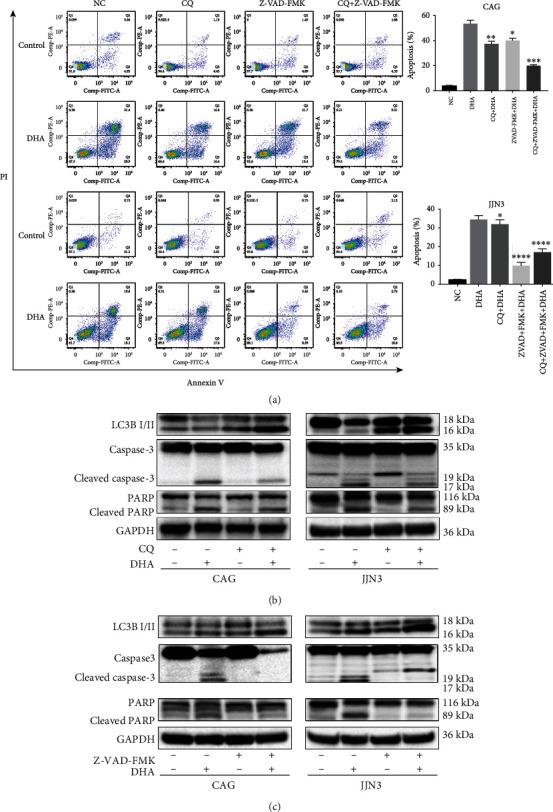
The connection between DHA-induced autophagy and apoptosis. (a) HMCLs were treated with 20 *μ*M DHA in combination with Z-VAD-FMK (100 mM), CQ (20 *μ*M), or both, and apoptosis was evaluated after 24 h by flow cytometry. The data are presented as the mean ± SD of three independent experiments. (b) HMCLs were preincubated with CQ (20 *μ*M) for 2 h, followed by treatment with the indicated concentrations of DHA for an additional 24 h. Whole-cell lysates were prepared for western blot analysis of LC3B, caspase-3, and PARP. (c) HMCLs were preincubated with Z-VAD-FMK (100 mM) for 2 h, followed by treatment with the indicated concentrations of DHA for an additional 24 h. Whole-cell lysates were prepared for western blot analysis of LC3B, caspase-3, and PARP. Data are presented as the mean ± SD of at least three independent experiments. ^∗^*P* < .05, ^∗∗^*P* < .01, ^∗∗∗^*P* < .001, ^∗∗∗∗^*P* < .0001.

In a previous article, DHA was found to induce autophagy, but this report did not assess the role of autophagy induced by DHA in MM cells [10]. Autophagy can either promote cell adaptation and survival or contribute to cell death [14, 15]. To determine whether DHA-induced autophagy has a prosurvival or prodeath effect, we used the autophagy inhibitor chloroquine (CQ), which can block autophagic flux and thus increase the level of LC3B-II, to pretreat the cells before DHA treatment. Annexin V-PI staining and flow cytometry were used to evaluate apoptosis. As shown in Figure 3(a), treatment with CQ reversed the apoptotic effects of DHA. In addition, immunoblotting with an anti-caspase-3 mAb revealed that the cleaved form of caspase-3 was significantly decreased after cotreatment with DHA and CQ for 24 h (Figure 3(b)). These findings show that DHA-induced autophagy plays a death-promoting role in MM cells.

As indicated above, the boundaries between apoptosis and autophagy are not completely clear, and crosstalk exists [16]. To further study the relationships between DHA-induced autophagy and apoptosis, we incubated the MM cells with an autophagy inhibitor and a caspase inhibitor.

First, we treated MM cells with the pan-caspase inhibitor Z-VAD-FMK during DHA treatment. As shown in Figure 3(a), we cultured MM cells with Z-VAD-FMK with/without 20 *μ*M DHA. The results showed that apoptosis induction by DHA was partially attenuated in CAG cells. In addition, the treatment of JJN3 cells with Z-VAD-FMK and DHA obviously inhibited DHA-induced cell death, although some of the cells were still alive. Z-VAD-FMK treatment alone at these concentrations did not show any cytotoxic effect during 24 h of exposure (Figure 3(a)). This finding suggests that DHA triggers caspase-dependent and caspase-independent cell death.

Since the treatment of MM cells with DHA induces caspase-dependent cell death, we further determined whether caspases modulate DHA-induced autophagy. We treated MM cells with DHA with/without 100 *μ*M Z-VAD-FMK prior to the addition of DHA. As expected, Z-VAD-FMK significantly inhibited the accumulation of activated caspase-3 (Figure 3(c)). Notably, blocking apoptosis with Z-VAD-FMK significantly increased the level of autophagosome formation as estimated by LC3B accumulation (Figure 3(c)).

To further examine the combined effects of CQ and Z-VAD-FMK treatment, HMCLs were pretreated with CQ and Z-VAD-FMK prior to DHA exposure, and the cells were stained with Annexin V and PI separately. As shown in Figure 3(a), when both autophagy and apoptosis inhibitors were used together, the number of apoptotic CAG cells further decreased, but no obvious decrease was observed in JJN3 cells.

### 3.4. P38/MAPK Signaling Pathway Is Responsible for Decreased Autophagy and Increased Apoptosis

**Figure 4:**
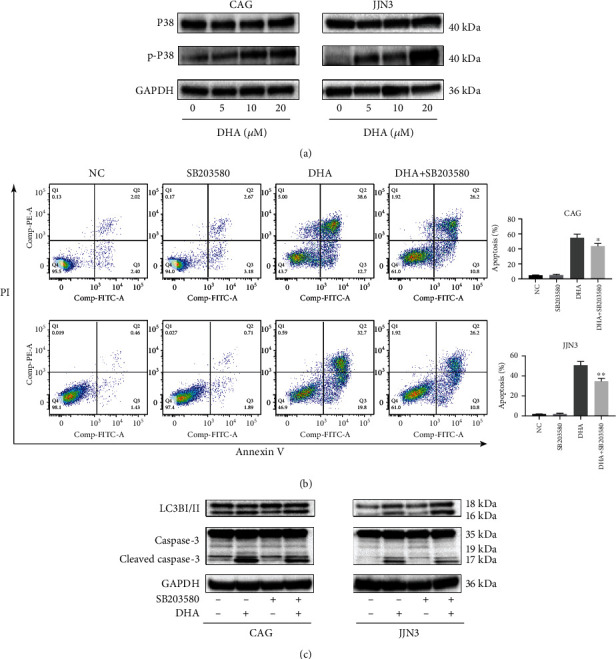
P38/MAPK signaling pathway is responsible for decreased autophagy and increased apoptosis. (a) HMCLs were treated with DHA at the indicated concentrations for 24 h. Cell extracts were analyzed by western blot for P38 phosphorylation and total P38 expression. (b) HMCLs were pretreated with SB203580 or not for 2 h and then incubated with 20 *μ*M DHA in the cell culture medium for 24 h. Apoptosis was evaluated after 24 h by flow cytometry. The data are presented as the mean ± SD (*n* ≥ 3). (c) HMCLs were pretreated with SB203580 or not for 2 h and then incubated with 20 *μ*M DHA in the cell culture medium for 24 h. Cell extracts were analyzed by western blot for LC3B and caspase-3. The summarized results are from at least three independent experiments. Values are presented as the mean ± SD. ^∗^*P* < .05, ^∗∗^*P* < .01, ^∗∗∗^*P* < .001. Data are presented as the mean ± SD of at least three independent experiments. ^∗^*P* < .05, ^∗∗^*P* < .01, ^∗∗∗^*P* < .001.

Notably, accumulating evidence suggests that MAPKs play important roles in cell proliferation, survival, and death [17, 18]. We next examined whether P38/MAPK is responsible for DHA-induced apoptosis in HMCLs. The results showed that DHA obviously increased the level of phospho-P38. Moreover, the expression levels of total P38 did not change (Figure 4(a)). These results indicate that DHA can stimulate the activation of P38/MAPK.

Since P38/MAPK has been reported to play a crucial role in the regulation of autophagy [19], we tested whether the activation of the P38/MAPK pathway plays a role in DHA-induced apoptosis and autophagy. We pretreated HMCLs with SB203580, a selective P38 inhibitor. Flow cytometric analysis showed that the combined DHA and SB203580 treatment could partially reverse the cell death induced by DHA (Figure 4(b)). Moreover, western blot analysis showed that the prior inhibition of P38 signaling followed by DHA treatment resulted in decreased caspase-3 activity and increased LC3B activity compared to DHA treatment alone (Figure 4(c)). All of the above results suggest that DHA-induced apoptosis and autophagy in HMCLs could be mediated by the P38/MAPK pathway.

### 3.5. DHA Inhibits MM Cell Proliferation and Promotes Autophagy via the Wnt/*β*-Catenin Pathway

**Figure 5:**
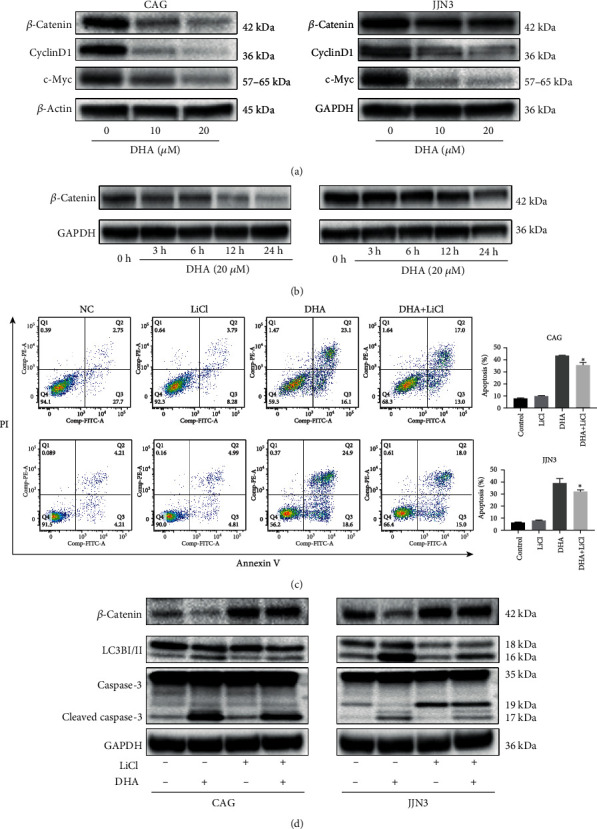
DHA inhibits MM cell proliferation and promotes autophagy via the Wnt/*β*-catenin pathway. (a) HMCLs were treated with DHA at the indicated concentrations for 24 h. Cell extracts were analyzed by western blot using *β*-catenin, Cyclin D1, and c-Myc antibodies. (b) HMCLs treated with 20 *μ*M DHA for the indicated times were analyzed by western blot for *β*-catenin expression. (c) HMCLs were treated with 20 *μ*M DHA in combination with LiCl or not, and apoptosis was evaluated after 24 h by flow cytometry. The data are presented as the mean ± SD (*n* ≥ 3). (d) Cells were incubated with DHA in the absence or presence of LiCl. Cell extracts were analyzed by western blot for *β*-catenin, LC3B, and caspase-3. Data are presented as the mean ± SD of at least three independent experiments. ^∗^*P* < .05, ^∗∗^*P* < .01, ^∗∗∗^*P* < .001.

Recent studies have shown that aberrant canonical Wnt signaling mediates the proliferation, migration, and drug resistance of MM cells [20]. To investigate the mechanism by which DHA induces apoptosis and autophagy, we treated MM cells with different concentrations of DHA. We observed that treatment with DHA for 24 h significantly reduced the protein levels of *β*-catenin and the target genes Cyclin D1 and c-Myc in a dose-dependent manner (Figure 5(a)). We also observed that treatment with DHA for 24 h reduced the protein levels of *β*-catenin in a time-dependent manner (Figure 5(b)). These results demonstrate that the antitumor effect of DHA is mediated by the suppression of gene downstream of Wnt/*β*-catenin. Next, we treated the cells with the Wnt agonist LiCl, which acts as a specific inhibitor of GSK3 and mimics Wnt signaling [21]. Treatment with LiCl alone substantially increased the levels of total *β*-catenin in the HMCLs, as shown by immunoblotting (Figure 5(d)). The cell viability was not significantly affected by 24 h of LiCl treatment, as demonstrated by flow cytometry (Figure 5(c)). The flow cytometric results showed that compared with DHA treatment alone, the combined treatment with DHA and LiCl reduced apoptosis (Figure 5(c)). Moreover, western blot analysis showed that treatment with DHA and LiCl reduced the protein levels of cleaved caspase-3 and LC3B compared with those of the control group (Figure 5(d)). Taken together, these data indicate that DHA inhibits proliferation and promotes autophagy via the Wnt/*β*-catenin signaling pathway in MM cells.

### 3.6. DHA Shows Antitumor Efficacy In Vivo

**Figure 6:**
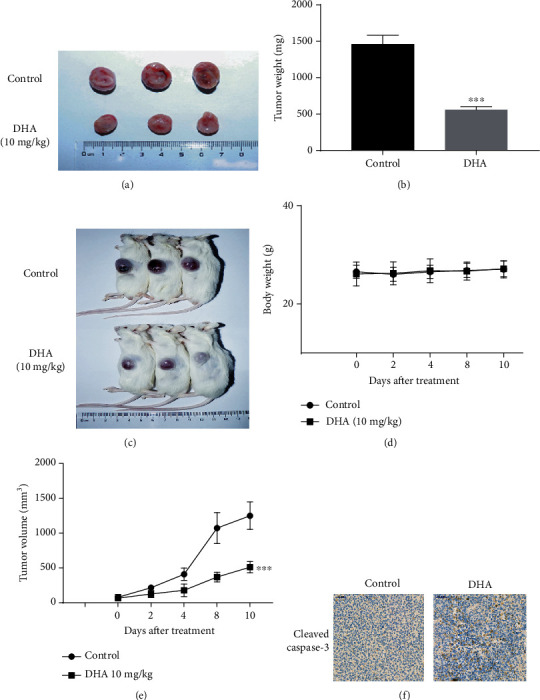
DHA shows antitumor efficacy in vivo. (a) Mice were killed, and tumors from the two groups are shown. (b) Tumor weight was measured. (c) Mice were killed, and tumors from the two groups are shown. (d) Mouse body weight was measured. (e) Tumor volumes were measured at the indicated time points. Representative data from three independent experiments are shown. (f) Immunofluorescence staining was performed to investigate cleaved caspase-3 expression in tumor tissues. The data are presented as the mean ± SD. ^∗^*P* < .05, ^∗∗^*P* < .01, ^∗∗∗^*P* < .001.

A xenograft mouse model was employed to further validate whether DHA treatment could inhibit growth in vivo. Human NOD-SCID-MM mice were subcutaneously injected with CAG cells and then treated with DHA or vehicle as a control. As shown in Figure 6(d), no significant body weight changes were observed after treatment with 10 mg/kg DHA, while a significant decrease in tumor volume was observed in the DHA-treated NOD/SCID mice compared with the vehicle-treated mice (Figure 6(a), Figure 6(c), and Figure 6(e)). In addition, the tumor weight was significantly reduced (Figure 6(b)). We next validated the expression of cleaved caspase-3 by immunohistochemistry. As expected, DHA treatment increased the expression of cleaved caspase-3 (Figure 6(f)). Collectively, these findings demonstrate that DHA has a marked tumor inhibition effect in vivo.

## 4. Discussion

Macroautophagy is a membrane-trafficking process that delivers cytoplasmic contents to lysosomes for degradation [12]. Under certain circumstances, autophagy acts as a self-limiting survival pathway, whereas in other cases, it constitutes an alternative cell death pathway [22]. However, the question of whether autophagy plays a role during DHA treatment has not been answered unequivocally. Hence, to study the role of DHA-induced autophagy and the mechanisms underlying the apoptosis and autophagy, we carried out the current studies.

In the present study, we found that both autophagy and apoptosis are responsible for DHA-induced cell death, as has also been described in *Drosophila* and sympathetic neurons in previous reports [23, 24]. These results prompted us to investigate the critical molecular mechanisms that trigger this phenomenon. It has been proposed that the activation of P53, a quintessential tumor suppressor and apoptosis inducer, inhibits mTOR activity and regulates autophagy, a tumor suppression process [25]. The autophagy-inducing activity of beclin-1 is inhibited by BCL2-family proteins such as Bcl-xl, which likely act as antiautophagic proteins via physical interactions with beclin-1 [26]. Our data may be explained by the latter studies, as treatment with DHA downregulated Bcl-xl expression. However, the explanation of those needs to be further verified.

The functional relationship between apoptosis and autophagy is complex, and we made three interesting observations in the inhibitor experiments in the present study. First, CQ pretreat was found to reduce the levels of cleaved caspase-3 in HMCLs, suggesting that in this model, autophagy precedes caspase-3-dependent apoptosis. Several studies have also shown that autophagy is required for apoptosis in certain circumstances [27, 28]. However, another study showed that although the initial phase of autophagy might be required, it is not sufficient to trigger apoptosis [29].

Second, Z-VAD-FMK pretreatment increased the accumulation of LC3B in DHA-treated cells, suggesting that autophagy increases when apoptotic cell death pathways are blocked. Regulation of autophagic activity by caspase has been noted in a previous study [30]. This finding suggested that inactivation of caspase-8 induces autophagy-related cell death. Hence, our findings, together with these results, support the hypothesis that autophagy is increased when apoptosis is blocked.

Third, in the presence of the two inhibitors CQ and Z-VAD-FMK, CAG cell death was further decreased by DHA treatment. These data suggest that autophagy and caspases can independently contribute to cell death. It is intriguing that some apoptosis features differed between CAG cells and JJN3 cells. We demonstrated herein that the treatment of JJN3 cells with CQ and Z-VAD-FMK did not cause a greater reduction in cell death than treatment with Z-VAD-FMK alone, suggesting that autophagy and apoptosis are successive events in the process of cell death. It is still unclear why the effects in the two cell lines are different. The effect could depend on the cell type, and the underlying mechanisms require further exploration.

P38/MAPK plays important roles in the execution of apoptosis and the regulation of cell survival and differentiation [31]. Recent studies have shown that many chemotherapeutic agents require P38 activity for the induction of cancer cell death [32–34]. Similarly, in the present study, DHA treatment dose-dependently activated P38/MAPK, which is necessary for apoptosis.

In addition to regulating apoptosis, P38/MAPK can also regulate autophagy. Emerging data suggest an inverse correlation between P38/MAPK pathway activation and autophagy [35, 36]. Our results showing that DHA can activate the P38/MAPK pathway, which leads to reduced autophagy, are consistent with those of previous studies. Moreover, more extensive investigations of P38/MAPK and its specific functions in apoptosis and autophagy are needed.

The Wnt/*β*-catenin pathway has emerged as one of the most frequently dysregulated signaling pathways in the majority of tumors [37], and the central mediator *β*-catenin is an essential transcriptional coregulator in the canonical Wnt pathway that forms complexes with the T cell-specific transcription factor/lymphoid enhancer-binding factor 1 (TCF/LEF) family of transcription factors to alter target gene expression and then promote cell proliferation and facilitate tumorigenesis [38, 39]. In this study, we found that DHA-induced cell death occurred through the inhibition of *β*-catenin expression.

The *β*-catenin signaling pathway is closely associated with autophagy. It has been shown that autophagy attenuates canonical Wnt signaling by enhancing disheveled degradation [40]. Previous findings revealed a regulatory feedback mechanism in which *β*-catenin negatively regulates autophagy, and *β*-catenin is itself targeted for autophagic clearances upon autophagic induction [41]. Consistent with these studies, we found that the activation of *β*-catenin by LiCl impaired autophagy induced by DHA in MM cells, suggesting that *β*-catenin plays a critical role in DHA-induced autophagy. It seems contradictory that P38 MAPK inhibits autophagy while inhibition of *β*-catenin can induce autophagy upon DHA exposure. We consider that cells in response to external stimuli is a complex and mutually regulated process, and *β*-catenin may play a more important role in DNA-induced autophagy.

**Figure 7:**
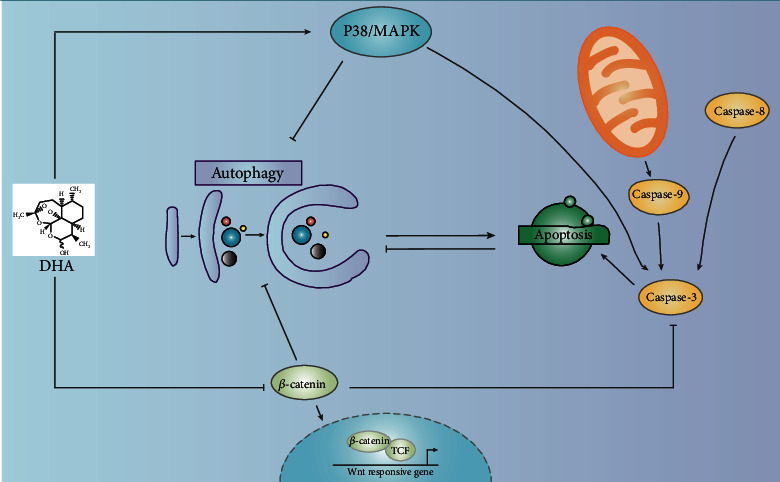
Schematic illustration of the molecular mechanism of DHA in apoptosis and autophagy in HMCLs.

In summary, we made key findings in this study. First, DHA treatment significantly induces cell death in a dose- and time-dependent manner via the extrinsic and intrinsic apoptosis pathways. Second, DHA-induced autophagy, which plays a prodeath role in MM, can regulate canonical apoptosis and vice versa. Finally, DHA modulates apoptosis and autophagy through the P38/MAPK signaling pathway and Wnt/*β*-catenin signaling pathway (Figure 7).

## Data Availability

The data used to support the findings of this study are available from the corresponding author upon request.
